# Respective roles of social deprivation, health literacy, and clinical factors for COVID-19: a case-control study in hospitalized patients

**DOI:** 10.3389/fpubh.2023.1239041

**Published:** 2023-11-21

**Authors:** Lotfi Dahmane, Chantal Julia, Nicolas Vignier, Lucile Sesé, Ségolène Brichler, Ruben Benaïnous, Hélène Bihan, Marilucy Lopez-Sublet, Damien Trawale, Olivier Bouchaud, Jeanne Goupil de Bouillé, on behalf of the SOCIALCOV group

**Affiliations:** ^1^Department of Infectious and Tropical Diseases, Hôpital Avicenne, Hôpitaux Universitaires Paris Seine-Saint-Denis, AP-HP, Bobigny, France; ^2^Public Health Department, GHU Paris-Seine-Saint-Denis, APHP, Bobigny, France; ^3^Centre de recherche en épidémiologie et statistiques sorbonne Paris cité (CRESS), Inserm, Inra, Cnam, University, Equipe de recherche en épidémiologie nutritionnelle (EREN), Bobigny, France; ^4^Centre d’Investigation Clinique Antilles Guyane, CIC INSERM 1424, Centre hospitalier de Cayenne, Cayenne, France; ^5^IAME, INSERM UMR 1137, Université Paris Cité, Université Sorbonne Paris Nord, UFR SMBH, Bobigny, France; ^6^French Collaborative Institute on Migration, Institut Convergences Migrations, ICM, Aubervilliers, France; ^7^Department of Pneumology, Hôpital Avicenne, Hôpitaux Universitaires Paris Seine-Saint-Denis, AP-HP, Bobigny, France; ^8^Department of Virology, Hôpital Avicenne, Hôpitaux Universitaires Paris Seine-Saint-Denis, AP-HP, Bobigny, France; ^9^Department of Intern Medicine, Hôpital Avicenne, Hôpitaux Universitaires Paris Seine-Saint-Denis, AP-HP, Bobigny, France; ^10^Department of Endocrinology, Hôpital Avicenne, Hôpitaux Universitaires Paris Seine-Saint-Denis, AP-HP, Bobigny, France; ^11^Department of Intern Medicine, AP-HP, CHU Avicenne, Centre d’Excellence ESH en Hypertension Artérielle, Bobigny, France; ^12^INSERM UMR 942 MASCOT, Paris 13-Univrsité Paris Nord, Bobigny, France; ^13^FCRIN INI-CRCT (Cardiovascular and Renal Clinical Trialists), Nancy, France; ^14^INED, Institut National d’études démographiques, Aubervilliers, France; ^15^Laboratoire Éducations et Promotion de la Santé, Université Sorbonne Paris Nord, LEPS, Bobigny, France

**Keywords:** social deprivation, COVID-19, hospital, health literacy, inequalities in health

## Abstract

**Introduction:**

To investigate the association between social deprivation and COVID-19 among hospitalized patients in an underprivileged department of the greater Paris area.

**Methods:**

Individuals hospitalized for COVID-19 between March 1st and October 31, 2020, were included, matched on age and sex, and compared with patients hospitalized for any other reason with negative RT-PCR for SARS-CoV-2, through a case-control study. Clinical, socio-demographic characteristics, health literacy, and social deprivation, assessed by the EPICES score, were collected. Factors associated with COVID-19 in hospitalized patients were assessed using univariate and multivariate logistic regression models.

**Results:**

69 cases and 180 controls were included. Participants were mostly men (*N* = 148: 59.4%) aged 65 or older (*N* = 109: 44.1%). Median EPICES score was 43.2 (IQR 29.4–62.9). EPICES score > 30.17 (precariousness threshold) was not significantly associated with COVID-19 in hospitalized patients (adjusted odds ratio (aOR) = 0.46; 95% Confidence Interval (CI) [0.21–1.01]). Advanced age, higher BMI, professional activity, home area of less than 25 m^2^ per person, and low health literacy, were significantly associated with COVID-19 in hospitalized patients.

**Discussion:**

This study highlights probable risk factors for specific exposition in disadvantaged area: maintenance of professional activity, smaller home area, and low health literacy.

## Introduction

SARS-CoV2 pandemic has caused nearly 7 million deaths worldwide to May 31, 2023 (1). Since its appearance, numerous studies have established clinical risk factors for infection, severity, and mortality to identify patients at risk to optimize management (2, 3). These risk factors vary depending on the outcomes studied, but for all the adverse outcomes for COVID-19, they include age, diabetes, hypertension, and overweight or obesity (4). Some studies have tried to establish a link between clinical risk for COVID-19 and socio-demographic data such as social deprivation, but these mostly used aggregate data, not allowing to study precisely the link between social deprivation at the individual level and the different outcomes of COVID-19 (4, 5). During the first wave, France, especially Paris and its suburbs, were particularly affected. France was ranked 3rd regarding the absolute number of deaths, after Italy and United Kingdom, from March 17 to April 29, 2020 (6). From March 2 to May 31, 2020, in France, all-cause mortality increased with +25,027 deaths compared to the expected number of deaths (7). Île-de-France (Paris and its suburbs) totalized the highest excess of deaths compared to other regions. In this area, the excess mortality from any cause from March 1 to April 30, 2020, was +124% in Seine-Saint-Denis department and +69.1% in Paris department (8). Yet, in Seine-Saint-Denis, the population is among the youngest in France but also one of the most socially deprived (9). In 1998, the French High Council for Public Health defined social deprivation as a social instability characterized by the loss of one or more of the securities, in particular that of employment, allowing individuals and families to assume their professional, family and social responsibilities and to enjoy their fundamental rights (10). It is related to employment, family activities, integration into the community, formal participation in social institutions, recreation and education (11). This is an important and yet understudied topic. Evidence strongly suggests an association between socioeconomic status and poorer various health outcomes, including infectious diseases mortality. Social deprivation depicts more specificities than socioeconomic status, as it is multidimensional and covers different aspects such as housing, social environment, and health care coverage. The *Evaluation de la précarité et des inégalités de santé dans les centres d’examens de santé* (EPICES) score is particularly used in France (12, 13). This score was first published in 2002 and is composed of 11 questions allowing to assess multiple dimensions of social deprivation (14). Health literacy is defined as the ability of an individual to gain access to, understand and use the information in ways to promote and maintain good health (15, 16). As social deprivation, it is a major topic and it is associated with poorer health outcomes (17). Data on the link between health literacy and COVID-19 adverse outcomes remain scarce, and further studies are highly required. The hypothesis of the present study was that social deprivation was associated with COVID-19 in hospitalized people. The main objective is to assess the link between social deprivation and COVID-19 disease in an underprivileged department of the greater Paris area. The secondary objectives explore the link between health literacy, clinical factors, and COVID-19.

## Materials and methods

### Study population and design

The SOCIALCOV study is a case-control study conducted at Avicenne University Hospital, Seine-Saint-Denis department, during the period from March 1 to October 31, 2020.

Inclusion criteria for participation in this study were to be older than 18 years, to consent to participate, and to be hospitalized during the study period.

Exclusion criteria were age less than or equal to 18 years, refusal to participate, cognitive impairment, to be institutionalized, healthcare professionals, to be hospitalized within the previous 15 days, being at the end of life, or on respiratory support other than nasal oxygen.

Patients were included as cases if they were hospitalized for COVID-19. COVID-19 was defined by a suggestive symptomatology with a positive Reverse Transcriptase Polymerase Chain Reaction (RT-PCR) for SARS-CoV2 and/or a CT scan considered as very likely by a radiologist. Controls were patients hospitalized for any other medical or surgical cause, with negative RT-PCR, no evidence of COVID-19 disease clinically or radiologically, and no exclusion criteria. Participants with COVID-19 disease were identified in the COVID unit during the epidemic period. Control patients were identified through a daily listing edited by the hospital virology department collecting all negative PCRs for SARS-CoV2.

Cases and controls were matched on age (10 years period) and sex. The recruitment of controls according to age and sex was thus adapted every 5 days according to the sex and age of the previously recruited cases. A minimum of two controls per case was required and additional controls were kept for statistical analysis as they were considered as additive information.

### Study size

In the absence of specific data on the association between social deprivation and COVID-19, we hypothesized a difference between cases and controls of 20% (35% of socially deprived cases and 15% for controls). These percentages were extrapolated using two studies using the EPICES score and conducted in the same center (13, 18). For a statistical power of 90% with a 5% alpha risk, the estimated number of subjects required was 92 cases and 184 controls, each case being matched with 2 controls.

### Data collection

After asking for consent and inclusion in the study, clinical study technicians and trained interviewers asked directly the questions to the patients and filled the questionnaire. Interview for patients who did not speak French were conducted using the telephone interpreting service *Inter Service Migrants Interprétariat* (more than 185 languages available).

The questionnaire domains provided information on socio-demographic and clinical characteristics, medical and surgical history, and health literacy. Our main outcome was to determine a potential association between social deprivation, assessed by the EPICES score, with SARS-CoV2 infection in hospitalized patients. The other characteristics were explored as independent variables, in order to assess a potential association with SARS-CoV2 infection in hospitalized patients.

Socio-demographic data collected were age, sex, country of birth, professional activity, surface area per person at home. This variable was obtained by dividing the home area (initially a categorical variable, use of the median of each interval for its continuous use) by the number of persons living in the household. Items for calculating the EPICES score were also collected (see the Supplementary Table S1). The reliability of this score was assessed in a disadvantaged area of northern France (12). It is composed of 11 questions and assess social deprivation from 0 (the least deprived) to 100 (the most deprived). The threshold for social deprivation was defined at 30.17 and established in a large cohort study carried out by the *Centre technique d’appui et de formation des centres d’examens de santé* (CETAF: Technical Center of Support and Training for Health Centers) (14). The EPICES score domains cover couple situation, social worker follow-up, health insurance coverage, ownership of housing, financial difficulties, material and social support in case of financial difficulties, the practice of a physical activity, and the leisure activities such as shows and vacations. Health literacy level was assessed using the Single Item Literacy Screener (19). This score has been initially used to assess reading ability in general, and is now used to identify low health literacy level among patients (20). Subjects were asked if they need help for reading medical information, and should answer by: always (lowest health literacy level), often, sometimes, rarely, or never (best health literacy level). Clinical data (weight, height, body mass index, medical, and surgical history) were systematically retrieved through the electronic medical record.

### Statistical analysis

All statistical analyses were performed using R Studio 3.6.0 software.

The null hypothesis was that social deprivation was not associated with COVID-19 in hospitalized patients. Quantitative variables were expressed as median and interquartile range, and categorical variables as number and percentage. The Kruskal–Wallis test compared the two populations on quantitative variables and the Chi-square test (or Fisher exact test if necessary) compared them on categorical variables. All significance tests were two-sided and a value of *p* of <0.05 was considered statistically significant to reject the null hypothesis.

A multivariate model was developed to assess risk factors for COVID-19 in hospitalized patients. Variables in the multivariate model were selected based on their significance in univariate analysis, their consensus in the literature, and finally their sociodemographic or clinical relevance. We also added EPICES score and health literacy level, as there are no data available concerning the interplay with COVID-19 (19). When calculating univariate and multivariate ORs, several variables were binarized or grouped to increase statistical power. Results were presented as crude odds ratio (cOR) and aOR with 95% CIs. Measurement of collinearity was performed using the Rstudio 3.6.0 software package “vif” indicating the variance inflation factor (VIF). The VIF threshold above which variables were considered collinear was set at 5.

### Ethical aspects

The study was approved by the local ethics committee of the Avicenne Hospital (CLEA-2020-126).

## Results

**Figure 1:**
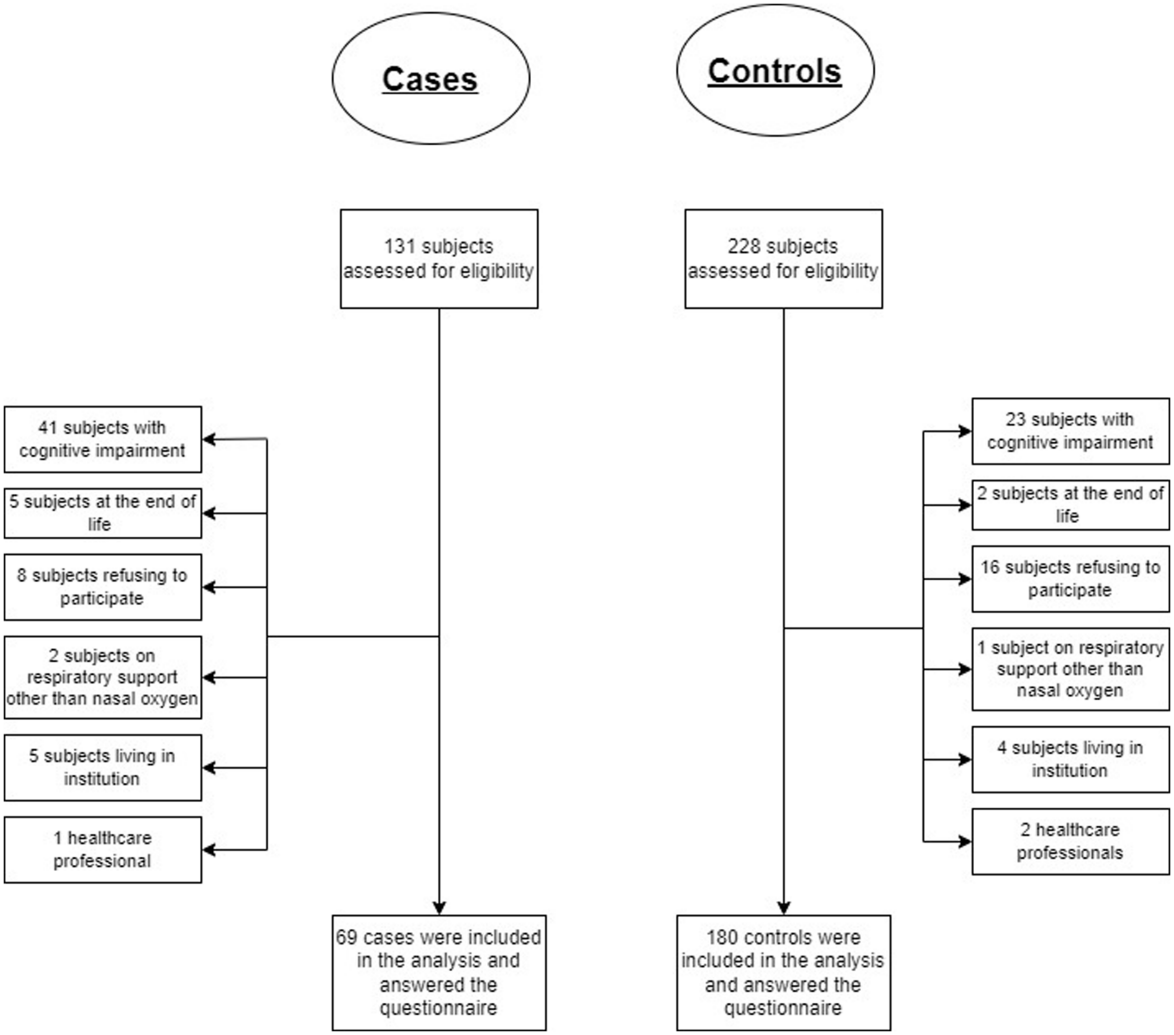
Study flowchart for cases and controls eligible for the study.

Between March 1 and October 31, 2020, 131 cases and 228 controls were eligible for the study. Sixty-two potential cases (41 patients with cognitive impairment, 5 patients at the end of life, 8 refusals to participate in the study, 2 patients on high-flow nasal oxygen therapy, 5 patients living in an institution, and 1 healthcare professional) and 48 potential controls (23 patients with cognitive impairment, 2 patients at the end of life, 16 refusals to participate in the study, 1 patient on high-flow nasal oxygen therapy, 4 patients living in an institution, and 2 healthcare professionals) were excluded. Analyses were therefore performed on 249 subjects included, 69 cases and 180 controls (see Figure 1).

Table 1 shows socio-demographic and clinical characteristics known as risk factors for COVID-19. For the population study, 109 (44.1%) were aged 65 years and older and 148 (59.4%) were men. There was no significant difference between the two groups regarding age and sex due to matching. Cases were more likely to report sharing their home with infected COVID-19 people (16.2% vs. 3.9%, *p* = 0.002). One hundred thirty-seven patients were overweight or obese (56.6%) and cases were more likely to be obese or overweight compared to controls (72.7% vs. 50.6%, *p* = 0.03). There were fewer smokers among cases population compared to controls (5.8% vs. 23.9%, *p* = 0.002).

**Table 1:** Socio-demographic and clinical characteristics for overall population, cases, and controls.

	Overall	Cases	Controls	Value of *p*
Age (years), *n* (%)	*N* = 247	*N* = 67	*N* = 180	0.89^a^
18–25	9 (3.6)	1 (1.5)	8 (4.4)	
26–35	16 (6.5)	3 (4.5)	13 (7.2)	
36–45	36 (14.6)	10 (14.9)	26 (14.4)	
45–54	29 (11.7)	7 (10.4)	22 (12.2)	
55–64	48 (19.4)	15 (22.4)	33 (18.3)	
65–74	62 (25.1)	19 (28.4)	43 (23.9)	
75+	47 (19)	12 (17.9)	35 (19.4)	
Sex, *n* (%)	*N* = 249	*N* = 69	*N* = 180	0.47^b^
Women	101 (40.6)	31 (44.9)	70 (38.9)	
Men	148 (59.4)	38 (55.1)	110 (61.1)	
Living with COVID-19 infected people	*N* = 247	*N* = 68	*N* = 179	0.002^a^
*N* (%)	18 (7.3)	11 (16.2)	7 (3.9)	
Hypertension	*N* = 249	*N* = 69	*N* = 180	0.58^b^
*N* (%)	99 (39.8)	25 (36.2)	74 (41.1)	
Diabetes	*N* = 249	*N* = 69	*N* = 180	1^b^
*N* (%)	75 (30.1)	21 (30.4)	54 (30)	
Overweight or obesity	*N* = 242	*N* = 66	*N* = 176	0.003^b^
*N* (%)	137 (56.6)	48 (72.7)	89 (50.6)	
Body Mass Index (kg/m^2^)	*N* = 242	*N* = 66	*N* = 176	<0.001^c^
Median (IQR)	26.4 (22.3–29.4)	27.8 (24.5–31)	25.1 (21.7–28.7)	
Smokers, *n* (%)	*N* = 249	*N* = 69	*N* = 180	0.002^b^
*N* (%)	47 (18.9)	4 (5.8)	43 (23.9)	
Drinking alcohol, *n* (%)	*N* = 249	*N* = 69	*N* = 180	0.34^b^
Less than once a day	58 (23.3)	14 (20.3)	44 (24.4)	
At least once a day	23 (9.2)	4 (5.8)	19 (10.6)	

a Fisher exact test.

b Chi-square test.

c Kruskal–Wallis test.

Table 2 shows socio-demographic, health literacy and social deprivation data explored in this study. Twenty-three cases (33.8%) were born in France while 78 (43.3%) were born in France among controls (*p* = 0.13). Among the 147 foreign-born patients, 34 (13.7%) were born in sub-Saharan Africa, and 56 (22.6%) were born in the Maghreb. Cases required more frequently an interpreter (23,1% vs. 9%, *p* = 0,0074). The health insurance coverage was not significantly different in the two groups. Cases were more often professionally active (39.1% vs. 22.2%) while controls were more often inactive for health reasons (0% vs. 11.7%, *p* = 0.003). The surface area per person living in the household was smaller in the cases (18.3 m^2^/person vs. 27.5 m^2^/person, *p* < 0.001). The level of health literacy between the two groups tended to be higher in the controls (*p* = 0.09). The median EPICES score for overall population was 43.2 (IQR 29.4–62.9). Social deprivation assessed by the EPICES score was 67.7% in cases and 77.1% in controls (*p* = 0.19).

**Table 2:** Exploratory characteristics for overall population, cases, and controls.

	Overall	Cases	Controls	Value of *p*
Country of birth, *n* (%)	*N* = 248	*N* = 68	*N* = 180	0.13^a^
Metropolitan France	101 (40.7)	23 (33.8)	78 (43.3)	
Overseas departments	9 (3.6)	1 (1.5)	8 (4.4)	
Sub-Saharan Africa	34 (13.7)	11 (16.2)	23 (12.8)	
Maghreb	56 (22.6)	14 (20.6)	42 (23.3)	
Asia	27 (10.9)	12 (17.6)	15 (8.3)	
America	4 (1.6)	1 (1.5)	3 (1.7)	
Europe	13 (5.2)	3 (4.4)	10 (5.6)	
Other	4 (1.6)	3 (4.4)	1 (0.6)	
Need for interpreter^d^	*N* = 242	*N* = 65	*N* = 177	0.0074^b^
*N* (%)	31 (12.8)	15 (23.1)	16 (9)	
Health insurance coverage, *n* (%)	*N* = 249	*N* = 69	*N* = 180	0.12^a^
Public^e^ and complementary^f^ insurance	154 (61.8)	35 (50.7)	119 (66.1)	
Public insurance without complementary insurance	33 (13.3)	9 (13)	24 (13.3)	
PUMa^g^ with CSS^h^	34 (13.7)	14 (20.3)	20 (11.1)	
PUMa without CSS	16 (6.4)	7 (10.1)	9 (5)	
AME^i^	4 (1.6)	2 (2.9)	2 (1.1)	
None	8 (3.2)	2 (2.9)	6 (3.3)	
Professional status, *n* (%)	*N* = 249	*N* = 69	*N* = 180	0.003^a^
Looking for a job	22 (8.8)	8 (11.6)	14 (7.8)	
Unemployed	25 (10)	7 (10.1)	18 (10)	
Active	67 (26.9)	27 (39.1)	40 (22.2)	
Student	7 (2.8)	1 (1.4)	6 (3.3)	
Inactive for health reasons	21 (8.4)	0 (0)	21 (11.7)	
Retired	107 (43)	26 (37.7)	81 (45)	
Surface area per person (m^2^/person)	*N* = 235	*N* = 68	*N* = 167	<0.001^c^
Median (IQR)	27.5 (16.63–42.1)	18.3 (13.75–27.5)	27.5 (18.3–42.5)	
<25, *n* (%)	112 (47.7)	42 (61.8)	70 (41.9)	0.0088^b^
25+, *n* (%)	123 (52.3)	26 (38.2)	97 (58.1)	
SILS^j^, *n* (%)	*N* = 237	*N* = 66	*N* = 171	0.09^a^
Never	155 (65.4)	37 (56.1)	118 (69)	
Rarely	14 (5.9)	2 (3)	12 (7)	
Sometimes	17 (7.2)	8 (12.1)	9 (5.3)	
Often	15 (6)	6 (9.1)	9 (5.3)	
Always	36 (15.2)	13 (19.7)	23 (13.5)	
EPICES Score	*N* = 244	*N* = 65	*N* = 179	0.53^c^
Median (IQR)	43.2 (29.4–62.9)	40.8 (27.2–62.7)	46.7 (30.2–63.02)	
EPICES Score, *n* (%)	*N* = 244	*N* = 65	*N* = 179	0.19^b^
≥30.17	182 (74.6)	44 (67.7)	138 (77.1)	
<30.17	62 (25.4)	21 (32.3)	41 (22.9)	

a Fisher exact test.

b Chi-square test.

c Kruskal–Wallis test.

d Need for the Inter Service Migrants Interprétariat service.

e Public health insurance, or National Health insurance is a health insurance that covers most of the health expenses for the majority of French individuals, working in France.

f Complementary insurance corresponds to a private insurance, covering the remaining costs of various health care.

g PUMa (Protection Universelle Maladie) is a state-guaranteed health coverage that covers health expenses for individuals who are not registered with the National Health insurance system, notably those who are unemployed or foreigners legally residing in France.

h CSS (Couverture Santé Solidaire) is a free complementary health insurance that covers the remaining costs of health care for people outside a certain income threshold who cannot afford to pay for a complementary insurance.

i AME (Aide Médicale d’Etat) is a health insurance that covers a certain part of health expenses for people who have stayed in France illegally for at least 6 months.

j Single Item Literacy Screener.

Table 3 shows EPICES score items. Cases appeared to be in couples more often (62.3% vs. 48.9%) but the difference was not significant (*p* = 0.079). Cases were more likely to have gone on vacation in the past 12 months (58.8% vs. 40%, *p* = 0.01).

**Table 3:** EPICES score items for overall population, cases, and controls.

	Overall	Cases	Controls	Value of *p*
Help from a social worker	*N* = 249	*N* = 69	*N* = 180	0.46^d^
*N* (%)	45 (18.1)	15 (21.7)	30 (16.7)	
Full health insurance	*N* = 249	*N* = 69	*N* = 180	0.39^d^
*N* (%)	188 (75.5)	49 (71)	139 (77.2)	
Living as a couple	*N* = 249	*N* = 69	*N* = 180	0.079^d^
*N* (%)	131 (52.6)	43 (62.3)	88 (48.9)	
Owner of his/her housing	*N* = 248	*N* = 68	*N* = 180	0.30^d^
*N* (%)	80 (32.3)	18 (26.5)	62 (34.4)	
Financial difficulties^a^	*N* = 248	*N* = 68	*N* = 180	0.72^d^
*N* (%)	85 (34.3)	25 (36.8)	60 (33.3)	
Sport activity^b^	*N* = 247	*N* = 67	*N* = 180	0.59^d^
*N* (%)	55 (22.3)	17 (25.4)	38 (21.1)	
Outing for shows^b^	*N* = 246	*N* = 66	*N* = 180	0.08^d^
*N* (%)	44 (17.9)	17 (25.8)	27 (15)	
Vacation^b^	*N* = 248	*N* = 68	*N* = 180	0.01^d^
*N* (%)	112 (45.2)	40 (58.8)	72 (40)	
Contact with relatives^c^	*N* = 248	*N* = 69	*N* = 179	1^d^
*N* (%)	137 (55.2)	38 (55.1)	99 (55.3)	
Help available for accommodation	*N* = 248	*N* = 68	*N* = 180	0.61^d^
*N* (%)	156 (62.9)	45 (66.2)	111 (61.7)	
Help available for material assistance	*N* = 249	*N* = 69	*N* = 180	1^d^
*N* (%)	164 (65.9)	45 (65.2)	119 (66.1)	

a Within the last month.

b Within the previous year.

c Within the last 6 months.

d Chi-square test.

Table 4 shows crude and adjusted OR for COVID-19 in hospitalized patients. In the multivariate model, subjects aged 65 to 74 years (aOR = 4.36; CI95% [1.39–14.99]) and older than 75 years (aOR = 3.88; CI95% [1.04–15.6]) were at greater risk for COVID-19 in hospitalized patients. Subjects born in sub-Saharan Africa or overseas departments were not at increased risk (aOR = 0.96; CI95% [0.37–2.36]). Being active professionally was a risk factor for COVID-19 after adjustment (aOR = 5.53; CI95% [1.84–18.38]) compared with being retired or inactive for health reasons. Job-seeking subjects appeared to be at higher risk as well, but the difference was not significant (aOR = 2.29; CI95% [0.74–7.41]). Subjects with low health literacy level were at higher risk compared with those with higher levels (aOR = 2.61; CI95% [1.22–5.68]). Being overweight or obese was also a risk factor for COVID-19 (aOR = 2.34; CI95% [1.17–4.86]). Social deprivation assessed by the EPICES score was not associated with COVID-19 in hospitalized patients (aOR = 0.47; CI95% [0.21–1.01]).

**Table 4:** Risk factors associated with COVID-19 in hospitalized patients.

	*N*	cOR (CI 95%)	Value of *p*	aOR (CI 95%) *N* = 214	Value of *p*
Age (years)	247				0.059^b^
Less than 55		1 (ref)		1 (ref)	
55–64		1.49 (0.68–3.26)	0.32	1.28 (0.48–3.39)	0.62
65–74		1.45 (0.70–3.01)	0.32	4.36 (1.39–14.99)	0.015
75+		1.13 (0.49–2.53)	0.78	3.88 (1.04–15.6)	0.048
Sex	249				
Women		1 (ref)		1 (ref)	
Men		0.78 (0.45–1.37)	0.39	0.70 (0.35–1.41)	0.32
Country of birth	248				
Other		1 (ref)		1 (ref)	
Sub-Saharan Africa or overseas departments		1.03 (0.48–2.1)	0.94	0.96 (0.37–2.36)	0.94
Professional status	249				0.008^b^
Retired or inactive for health reasons		1 (ref)		1 (ref)	
Unemployed, looking for a job or student		1.65 (0.79–3.40)	0.175	2.29 (0.74–7.41)	0.16
Active		2.65 (1.38–5.11)	0.003	5.53 (1.84–18.38)	0.003
Surface area per person (m^2^/person)	235				
25+		1 (ref)		1 (ref)	
<25		2.24 (1.26–4.03)	0.006	2.77 (1.35–5.87)	0.006
SILS^a^	237				
Never or rarely		1 (ref)		1 (ref)	
Sometimes, often or always		2.2 (1.2–4.02)	0.01	2.61 (1.22–5.68)	0.014
Overweight or obese	242				
No		1 (ref)		1 (ref)	
Yes		2.61 (1.43–4.93)	<0.01	2.34 (1.17–4.86)	0.02
EPICES score	244				
<30.17		1 (ref)		1 (ref)	
≥30.17		0.62 (0.33–1.18)	0.14	0.47 (0.21–1.01)	0.053

a Single Item Literacy Screener.

b Global value of *p* for the variables with more than 2 modalities.

## Discussion

In our case-control study in a hospitalized population, social deprivation assessed by the EPICES score was not associated with COVID-19 in hospitalized patients. Factors associated with COVID-19 were advanced age, being professionally active, surface area inferior to 25 m^2^ per person, low health literacy level, and being overweight or obese.

The lack of association between social deprivation assessed by the EPICES score and COVID-19 in hospitalized patients can be explained by several hypotheses. First, this score has not been evaluated for migrant populations, which was the majority in our study (59% of foreign-born persons). For example, the question asked about vacation during the last 12 months might not reflect social deprivation in the same way for migrant populations, who may return to their country of birth despite financial difficulties, compared to non-migrant populations. These limitations had already been highlighted by various authors (18). Second, a very high level of social deprivation within the two groups of our study population, and a strong homogeneity in the study population, making it difficult to identify significant differences (13). Third, some items in the score measuring social interactions (living as a couple, going on vacations, leisure activities) may be protective factors for social deprivation within the EPICES score, but risk factors for COVID-19 specifically. Indeed, there is considerable evidence that social gatherings are risk factors for transmission of SARS-CoV2 (21). Furthermore, Shah et al. show in a meta-analysis that the highest secondary attack rate within a household corresponds to husbands or wives (22).

Other dimensions of social deprivation not explored by the EPICES score are associated with high social interactions. Our study is consistent with the literature on the subject such as promiscuity in the household or the pursuit of so-called “essential” work which does not permit home confinement (23, 24). Through the concept of syndemics, many authors have underlined difficulties of weighting risk factors of social deprivation in the COVID-19 outbreaks (25). Our work also highlights the problem of its measurement tool.

Our study shows an unknown association between health literacy level when dichotomized, and COVID-19 in hospitalized patients, independent of social deprivation, as shown in multivariate analyses. Health literacy is linked to literacy and entails people’s knowledge, motivation, and competence to access, understand, appraise, and apply health information in order to make judgments and take decisions in everyday life concerning healthcare, disease prevention and health promotion to maintain or improve quality of life during the life course (26). Single Item Literacy Screener was used for assessing health literacy, which is a validated score in the literature, mostly for its simplicity and its reproducibility, as it consists of a single question: “How often do you need to have someone help you when you read instructions, pamphlets, or other written material from your doctor or pharmacy?.” This way of assessment was more convenient in a public health crisis, working in a COVID-19 unit. In order to minimize response bias related to comprehension of the question, we excluded patients with cognitive impairment and used an interpreter for non-French speakers. However, a social conformity bias, inherent to this type of survey, may have been present.

Do et al. showed that healthcare professionals with higher health literacy had fewer symptoms related to COVID-19, but no virological documentation was performed (27). Some studies have shown a correlation between good health literacy level and the application of barrier gestures (28, 29). Health literacy’s definition underlines the importance of various skills during a health crisis, not only in terms of understanding and applying barrier gestures, but also in terms of identifying symptoms requiring urgent care and following treatment recommendations. It is possible that subjects with higher levels of health literacy are more able to acquire information from public authorities or health care personnel regarding the barrier gestures to be applied to limit the risks of infection.

Our study also found already well-established risk factors, including body mass index. The association between overweight and COVID-19 is particularly strong, and the results of our study are consistent with multiple data in the literature (30). This was not the case for hypertension and diabetes. The association between COVID-19 and conditions such as diabetes and hypertension appear to be established. It is possible that the strength of this association may be less important than the association between COVID-19 and higher BMI (31).

Many studies have showed an excess risk of infection in black and Asian populations, which can be explained by greater social deprivation in these populations. Data are often collected in an aggregate fashion, and may lead to a confounding bias (32–34). Furthermore, the link with mortality may be less clear (35). Skin color was not collected because ethnic statistics are prohibited in France. No excess risk of infection was found among people born in sub-Saharan Africa and in the French overseas departments and territories. These results are consistent with another study conducted in Paris suburbs which showed no association between country of birth and COVID-19 severity (36). This suggests that the heterogeneity of the results in the literature may be explained by the different approach to collecting data on ethnicity.

Despite the significant difference regarding the need for an interpreter in our univariate analysis, we did not include this variable in our multivariate model, preferring to select the country of birth. In our population, some foreign-born subjects learnt French language because they were living in France for some time, or already learnt French language before their arrival. To be consistent with data in the literature that focus on ethnicity or skin color, we believe that some subjects with a non-white ethnicity would not have been analyzed properly.

Our study had several limitations. First, our population was particularly socially deprived in both groups, making it more difficult to identify a difference. Despite the high prevalence of precarious subjects, some data emerged as significant after adjustment, such as low health literacy level or household surface area per person. Second, our study was monocentric, limiting the recruitment pool. Third, our population was recruited between the months of March and October 2020, corresponding to heterogeneous periods in terms of lockdown policies, barrier gesture performance, mask availability, or local or national incidence.

Our study is original because it combines clinical with socio-demographic data using original case-control design. We assessed the multidimensional aspect of social deprivation without limiting ourselves to simple administrative criteria such as salary or health insurance coverage. This is also one of the first study to our knowledge to assess the relationship between health literacy level and documented COVID-19. This assessment was done using an easy-to-use and reproducible tool. Low health literacy level was only significantly associated with COVID-19 when dichotomized. Health literacy remains a complex topic and further studies on this subject seem highly required.

## Conclusion

In conclusion, these results highlight the difficulty in establishing the link between COVID-19 in hospitalized patients and social deprivation from data collected at the individual level and the difficulty of using and transposing scores in research. Other scores than EPICES have been used in other countries and they might not reflect the same dimensions of social deprivation. Moreover, these different scores can only be used in specific configurations, especially in terms of country, health system or specificities of various populations. However, thanks to an adjustment on socio-demographic and clinical data, our study makes it possible to free ourselves from certain possibly confounding factors. It makes it possible to determine the influence of certain factors such as precariousness, obesity, and level of literacy, in relation to COVID-19, while measuring their interdependence. However, it does not allow us to say whether these factors are cumulative, within the framework of a syndemic (37).

## Data availability statement

The raw data supporting the conclusions of this article will be made available by the authors, without undue reservation.

## Ethics statement

The studies involving humans were approved by Ethics committee of the Avicenne Hospital (CLEA-2020-126). The studies were conducted in accordance with the local legislation and institutional requirements. The participants provided their written informed consent to participate in this study.

## Author contributions

CJ, OB, and JG designed the study. CJ and JG developed the questionnaire. LD and JG managed the data collection, oversaw the data collection and maintained the database, performed the statistical analyses, and drafted the first versions of the manuscript. All authors contributed to the article and approved the submitted version.
