# Supplementary material for: Respective roles of social deprivation, health literacy, and clinical factors for COVID-19: a case-control study in hospitalized patients
Source: Front Public Health. 2023 Nov 21;11:1239041. doi: 10.3389/fpubh.2023.1239041 (PMC10702549; doi:10.3389/fpubh.2023.1239041)
Supplement: Supplementary file 1 [file Table_1.DOCX]

Table S1 – EPICES Score items and calculation

| Questions | Score | |
| --- | --- | --- |
|  | Yes | No |
|  |  |  |
| Do you sometimes meet with a social worker (welfare worker, educator)? | 10.06 | 0 |
| Do you have complementary health insurance (mutual insurance)? | -11.83 | 0 |
| Do you live as a couple? | -8.28 | 0 |
| Are you a homeowner or will you be one in the near future? | -8.28 | 0 |
| Are there periods in the month when you have real financial difficulties in facing you needs (food, rent, electricity)? | 14.80 | 0 |
| Have you participated in any sports activities in the last 12 months? | -6.51 | 0 |
| Have you gone to any shows (cinema, theatre) in the last 12 months? | -7.10 | 0 |
| Have you gone on holiday during the past 12 months? | -7.10 | 0 |
| Have you seen any family members in the past six months (other than your parents or children)? | -9.47 | 0 |
| Did you have difficulties (financial, family or health), is there anyone around you who could take you in for a few days? | -9.47 | 0 |
| Did you have difficulties (financial, family or health), is there anyone around you who could help you financially (material aid such as lending you money)? | -7.10 | 0 |
| Intercept | 75.14 | NA |

Calculation of EPICES Score: every question must be answered. The EPICES score is computed by adding each question coefficient to intercept whenever the answer is yes.
